# Six host range variants of the xenotropic/polytropic gammaretroviruses define determinants for entry in the XPR1 cell surface receptor

**DOI:** 10.1186/1742-4690-6-87

**Published:** 2009-10-07

**Authors:** Yuhe Yan, Qingping Liu, Christine A Kozak

**Affiliations:** 1 Laboratory of Molecular Microbiology, National Institute of Allergy and Infectious Diseases, Bethesda, MD 20892-0460, USA

## Abstract

**Background:**

The evolutionary interactions between retroviruses and their receptors result in adaptive selection of restriction variants that can allow natural populations to evade retrovirus infection. The mouse xenotropic/polytropic (X/PMV) gammaretroviruses rely on the XPR1 cell surface receptor for entry into host cells, and polymorphic variants of this receptor have been identified in different rodent species.

**Results:**

We screened a panel of X/PMVs for infectivity on rodent cells carrying 6 different XPR1 receptor variants. The X/PMVs included 5 well-characterized laboratory and wild mouse virus isolates as well as a novel cytopathic XMV-related virus, termed Cz524, isolated from an Eastern European wild mouse-derived strain, and XMRV, a xenotropic-like virus isolated from human prostate cancer. The 7 viruses define 6 distinct tropisms. Cz524 and another wild mouse isolate, CasE#1, have unique species tropisms. Among the PMVs, one Friend isolate is restricted by rat cells. Among the XMVs, two isolates, XMRV and AKR6, differ from other XMVs in their PMV-like restriction in hamster cells. We generated a set of *Xpr1 *mutants and chimeras, and identified critical amino acids in two extracellular loops (ECLs) that mediate entry of these different viruses, including 3 residues in ECL3 that are involved in PMV entry (E500, T507, and V508) and can also influence infectivity by AKR6 and Cz524.

**Conclusion:**

We used a set of natural variants and mutants of *Xpr1 *to define 6 distinct host range variants among naturally occurring X/PMVs (2 XMV variants, 2 PMVs, 2 different wild mouse variants). We identified critical amino acids in XPR1 that mediate entry of these viruses. These gammaretroviruses and their XPR1 receptor are thus highly functionally polymorphic, a consequence of the evolutionary pressures that favor both host resistance and virus escape mutants. This variation accounts for multiple naturally occurring virus resistance phenotypes and perhaps contributes to the widespread distribution of these viruses in rodent and non-rodent species.

## Background

Retroviruses enter cells through interaction with specific cell surface receptors. This virus-receptor interaction defines host range, contributes to pathogenesis, and can provide the basis for the evolution of restriction variants that enable natural populations to evade retrovirus infection. To date, six receptors for mouse gammaretroviruses have been identified. All six are transporters with multiple transmembrane domains, and five of the six are used by different host range subclasses of mouse leukemia viruses (MLVs) [1]. Two of these MLV receptors have naturally occurring variants associated with virus resistance: the CAT-1 receptor for the ecotropic (mouse-tropic) MLVs and the XPR1 receptor for the xenotropic and polytropic MLVs (XMVs, PMVs), viruses capable of infecting cells of non-rodent species. Studies on these receptors have identified residues critical for virus entry, and described 2 variants of CAT-1 and 4 variants of XPR1 in *Mus *species that differ in their ability to mediate entry of various virus isolates [2-7].

The four functionally distinct variants of the receptor gene, *Xpr1*, are found in different taxonomic groups of *Mus*. *Xpr1*^*n *^is found in European *M. m. domesticus*, and was originally described in the laboratory mouse [8-10]. *Xpr1*^*c *^is found in the Asian species *M. m. castaneus *[5]; *Xpr1*^*p *^is in the Asian species *M. pahari *[7]; and *Xpr1*^*sxv *^is in other Eurasian species [4]. These variants are distinguished by their differential susceptibility to prototype XMV and PMV viruses as well as to the wild mouse isolate, CasE#1 [7]. The XMV and PMV virus subgroups were initially defined by the ability of PMVs but not XMVs to infect cells of the laboratory mouse [11-13], and by the cytopathic and leukemogenic properties of PMVs, also termed MCF MLVs (mink cell focus-inducing MLVs). CasE#1 differs from the XMV and PMV subtypes in sequence and biological properties [7,14]. The observed host range differences of these virus isolates are due to sequence polymorphisms in both receptor and viral envelope genes.

The XPR1 receptor has 8 predicted transmembrane domains, and 4 extracellular loops (ECLs) [8-10]. Sequence comparisons and mutagenesis have identified independent receptor determinants in two of these loops, ECL3 and ECL4 [6,15]. Two critical amino acids have been defined for XMV entry, K500 in ECL3, and T582 in ECL4 [6,7]. These two receptor determinants independently produce XMV receptors but are not functionally equivalent; as the T582Δ insertion into *Xpr1*^*n *^generates a receptor for CasE#1, but the K500E substitution does not [7]. The receptor determinant for PMV has not been defined, although it was determined to be in ECL3 of *Xpr1*^*n *^but is independent of the ECL3 K500 XMV determinant [7].

In this study, we use a set of natural variants and mutants of *Xpr1 *to define 6 distinct host range variants among naturally occurring X/PMVs and to identify critical amino acids in XPR1 that mediate entry of these viruses. The 6 viruses include a novel cytopathic XMV-related virus, termed Cz524, isolated from an Eastern European wild mouse. Among the 5 previously described isolates, we define a variation in species tropism that distinguishes PMV isolates, and we demonstrate that one mouse XMV, AKR6 MLV, shares unusual host range properties with XMRV, a xenotropic-like virus isolated from human prostate cancer [16,17].

## Results

### Host range and sequence variations among X/PMVs

**Figure 1:**
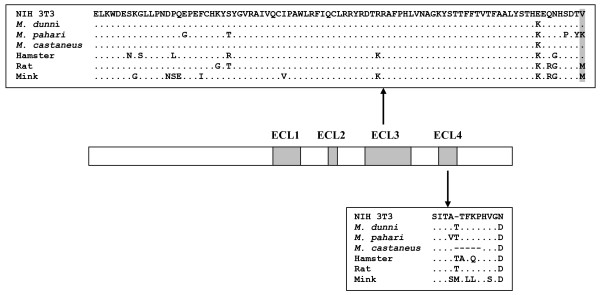
**Comparison of the deduced amino acid sequences of the ECL3 and ECL4 domains of the *Xpr1 *genes of rodents and mink**. Ferret XPR1 is identical to that of mink.

The X/PMV viruses of mice represent a highly polymorphic group. While most isolates have either XMV or PMV host range, several have been described with atypical species tropism [14,18]. To characterize host range variation within the X/PMVs, we screened a panel of X/PMVs along with amphotropic MLV (A-MLV) (Table 1) for infectivity in rodent cells with different XPR1 receptors (Fig. 1). In addition to 6 laboratory mouse virus isolates and 3 previously described wild mouse isolates, this panel included a novel isolate from the eastern European wild-mouse derived strain, CZECH/EiJ, and XMRV, a xenotropic-like virus isolated from human prostate cancer patients [16,17]. LacZ pseudotypes were generated for these viruses and tested for infectivity on mouse cells carrying the 4 known *Mus Xpr1 *variants, on rat and hamster cells, and on nonrestrictive mink lung cells.

**Table 1:** Viruses used in infectivity studies.

**MLV**		**Mouse**		
			
**Type**	**Virus**	**Strain/Species**	**Tissue/Cell**	**Reference**
PMV	FrMCF	NIH Swiss	Leukemic spleen of mouse inoculated with FrMLV	This report
	
	HIX MLV		IC strain of Moloney MLV grown in cat and Swiss mouse cells	[11]
	
	MCF 247	AKR	Thymus of 6 month old mouse	[12]

XMV	CAST-X	*M. castaneus*	IUdR/LPS treated spleen cells	[7]
	
	AKR6	AKR	Thymus of 2 month old mouse	[12]
	
	NZB-IU-6	NZB	IUdR treated embryo fibroblasts	[40]
	
	NFS-Th1	NFS	Thymus of a 5.5 month old mouse	[41]
	
	XMRV	human	Prostate cancer	[16,17]

X/PMV	CasE#1	Lake Casitas, California wild mouse	IUdR treated embryo cells	[14]

X/PMV	Cz524	CZECHII/ EiJ	Spleen of 2 month old inoculated with MoMLV	This report

A-MLV	4070A	Lake Casitas, California mouse	Embryo cells	[42]

### PMVs: a Friend PMV with novel tropism

The two PMV isolates showed the same pattern of infectivity on mouse cells carrying the 4 variants of *Xpr1 *(Table 2). Both viruses infected NIH 3T3 (*Xpr1*^*n*^) and cells carrying *Xpr1*^*sxv*^, but did not infect cells of *M. pahari *(*Xpr1*^*p*^) or cells carrying *Xpr1*^*c*^. Chinese hamster cells were resistant to both viruses. Rat2 cells, however, were efficiently infected by HIX PMV, but were very resistant to FrMCF (Table 2). The resistance to FrMCF was observed only with this particular Friend PMV isolate as Rat2 cells were efficiently infected by three other Friend MCF PMVs as well as by MCF 247 (not shown). Resistance to this FrMCF was also observed in rat XC cells (not shown) indicating that this resistance is not limited to the Rat2 cell line.

**Table 2:** Virus titers of X/PMV LacZ pseudotypes on rodent and mink cells carrying variants of the *Xpr1 *receptor.

		**Log_10 _LacZ Pseudotype Titer^a^**							
		
**Mouse** **Receptor**		**PMV**		**XMV**			**X/PMV**	**X/PMV**	
		
	**Cells**	**HIX**	**FrMCF**	**CAST-X**	**AKR6**	**XMRV**	**CasE#1**	**Cz524**	**A-MLV**
*Xpr1*^*n*^	NIH 3T3	5.2+/-0.3	5.1+/-0.3	0	0	0	0	0	5.2+/-0.5
	
*Xpr1*^*sxv*^	NXPR-S	4.3+/-0.1	4.3+/-0.4	3.5+/-0.4	3.7+/-0.4	1.2+/-0.5	2.4+/-0.2	4.8+/-1.1	5.2+/-1.1
	*M. dunni*	4.4+/-0.9	5.2+/-0.6	5.6+/-0.4	5.4+/-0.2	3.7+/-0.2	5.3+/-0.4	5.8+/-0.1	4.9+/-0.1
	
*Xpr1*^*c*^	NXPR-C	0	0	3.5+/-0.5	2.8+/-0.3	0.5+/-0.3	0	1.0+/-0.4	4.2+/-0.9
	
*Xpr1*^*p*^	*M. pahari*	0	0	4.7+/-0.3	4.5+/-0.4	3.3+/-0.3	4.5+/-0.4	0	3.9+/-0.4
	
	Hamster	0	0	1.1+/-0.5	0	0	0	0	3.7
	
	Rat	4.6+/-0.1	0.5+/-0.5	5.2+/-0.4	5.1+/-0.1	3.1+/-0.6	5.1+/-0.5	1.7+/-0.6	4.7+/-0.6
	
	Mink	5.5+/-0.3	5.6+/-0.1	5.3+/-0.3	5.1+/-0.3	4.2+/-0.4	5.1+/-0.3	5.0+/-0.6	4.5+/-0.9

^a^Measured as the number of cells positive for β-galactosidase activity in 100 ul of virus. Where no SD is given, infectivity was only tested once. 0, no positive cells in cultures infected at least 3 times with 0.1 ml of undiluted pseudotype stock.

**Figure 2:**
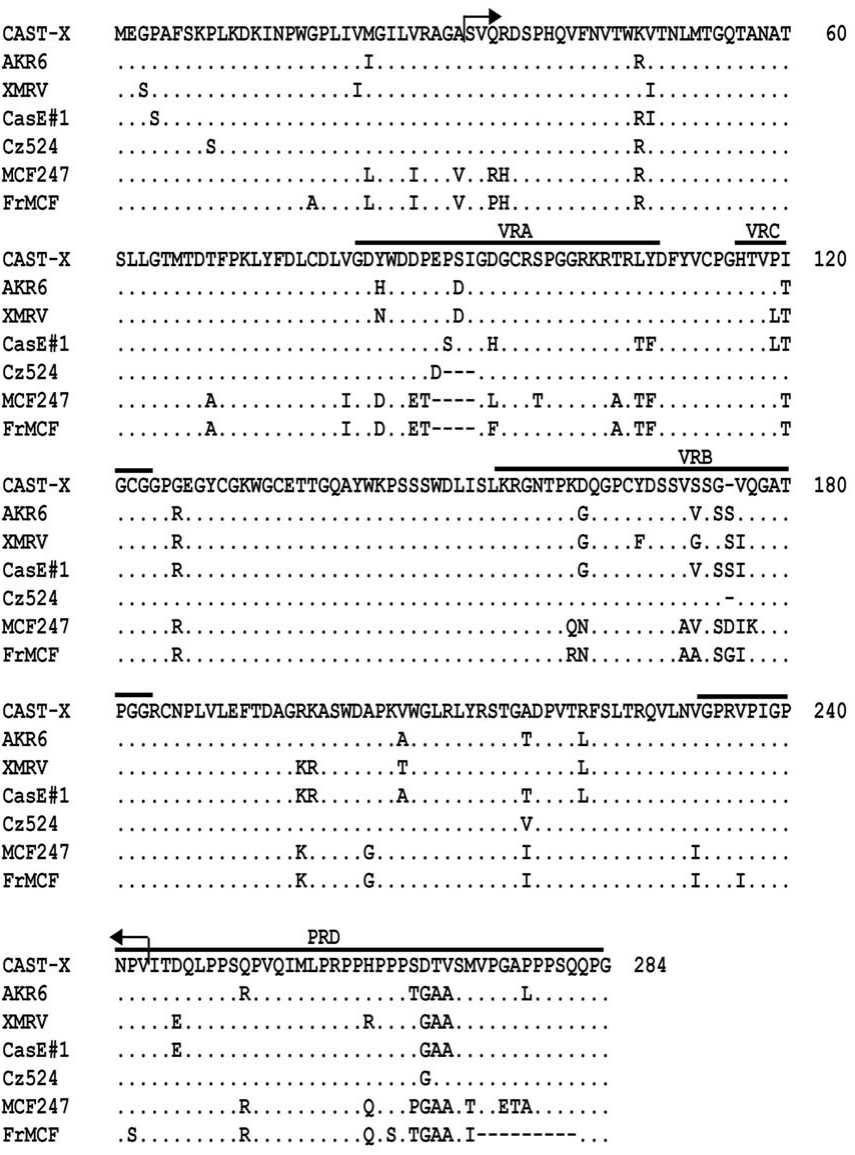
**Comparison of the deduced amino acids sequences of the RBD region of the viral *env *gene of the X/PMVs used for infection**. Variable regions VRA, VRB and VRC are indicated with bars. Arrows indicate the beginning and end of the SUenv RBD. Sequences for CAST-X, AKR6, XMRV, CasE#1, and MCF247 were previously determined (GenBank Nos. EF606902, DQ199948, EF185282, EF606901, K00526).

*Env *sequence comparisons identified scattered substitutions that distinguish FrMCF and other PMVs and the presence of a 9 codon deletion unique to FrMCF (Fig. 2). This deletion has been identified in few replication competent PMVs [19,20], although it is a hallmark of modified PMV-related endogenous *env *genes (Mpmvs) [21]. This deletion is outside the Env receptor binding domain (RBD) [22], and lies in the proline-rich domain (PRD), a region that is thought to mediate conformational changes in Env during infection and to influence membrane fusion [23].

### Cz524 MLV

In an attempt to recover novel PMV-type recombinant viruses, we inoculated mice of different taxonomic groups with MoMLV. Using this approach, we previously described a set of replication competent recombinant PMVs isolated from MoMLV inoculated *M. spretus *[24]. In the present study, we inoculated 11 CZECHII/EiJ mice, an inbred line of *M. m. musculus*. These mice carry dozens of XMV *env *genes, but few PMV copies [25], unlike the common strains of laboratory mice which carry multiple XMV and PMV endogenous *env *genes [21]. Spleen or thymus cells from 2 month old inoculated mice were plated on *M. dunni *and/or mink cells, and media collected from one of these *M. dunni *cultures induced MCF-type foci on mink cells (not shown). Southern blotting of virus infected cells with ~ 120 bp *env*-specific probes identified sequences related to XMVs, but no PMV *env*-related fragments (not shown). The virus was biologically cloned by limiting dilution, and its *env *gene was cloned and sequenced.

The sequenced Cz524 *env *was not an *env *recombinant derived from the inoculated MoMLV; no segments identical to MoMLV were identified although the breakpoint positions identified in other MoMLV recombinants cluster in an *env *region just downstream of PRD [19]. Consistent with the Southern blot analysis, the *env *sequence of Cz524 MLV showed closest homology to XMVs (Fig. 2). Of the 33 RBD amino acid residues that distinguish Cz524 from MCF 247 PMV or CAST-X XMV, Cz524 resembled the prototype XMV at 26 sites, the prototype PMV at 4 sites, and had novel residues at 3 sites. The major difference between Cz524 and XMV viruses is in VRA, the first variable domain in SUenv, where PMVs have a 4 codon deletion relative to XMVs. Cz524 has a 3 codon deletion relative to XMVs at this same position, and there is a novel substitution at the 4^th ^site typically deleted in PMVs.

LacZ pseudotypes carrying the Cz524 Env were tested for infectivity on rodent and mink cells (Table 2). Cz524 shows a novel pattern of species tropism that differs from that of CasE#1 and all XMVs and PMVs tested. This virus infects mink cells and cells carrying *Xpr1*^*sxv *^with high efficiency, shows very poor infectivity on cells carrying *Xpr1*^*c *^and on Rat2 cells, and is restricted by hamster cells and cells carrying the mouse *Xpr1*^*n *^and *Xpr1*^*p *^variants.

### XMVs: a host range variant defined by AKR6 and XMRV

Three of the four XPR1 variants of *Mus *supported replication of XMVs; only *Xpr1*^*n *^of the laboratory mouse strains failed to mediate infection of any of these viruses (Table 2). Among the susceptible mouse cells, there was variation in infectivity by the 3 XMVs, and this could be due to receptor polymorphism or non-receptor factors. The pseudotypes that we used here carry the Gag proteins of their parental viruses, and studies on some XMVs [26] indicates that they may be subject to restriction by *Fv1*, a mouse gene responsible for post-entry virus resistance that targets specific capsid residues. The capsid sequence for one of the 3 XMVs used in this analysis, XMRV, has been determined [16], and it carries the *Fv1*^*n *^target residue E110 [27]. The NXPR-S and NXPR-C cells carrying *Xpr1*^*sxv *^and *Xpr1*^*c *^have the restrictive *Fv1*^*n *^allele. Therefore, to determine if our XMV pseudotypes are subject to *Fv1 *restriction, we examined infectivity in a second cell line carrying *Xpr1*^*sxv*^, the *Fv1-*null *M. dunni *cell line (Table 2). We noted an *Fv1*-type 100-1000 fold reduction in infectivity of all 3 XMVs in NXPR-S relative to *M. dunni*. A similar 1000-fold reduction for CAST-X was observed in NFS/N cells carrying *Xpr1*^*c*^, but infectivity with XMRV and AKR6 was further reduced in these cells, suggesting either that this XPR1 variant is not an efficient receptor for these particular XMV viruses, or that additional factors inhibit infection. These observations taken together indicate that while there are some infectivity differences that are consistent with *Fv1 *restriction, both *Xpr1*^*sxv *^and *Xpr1*^*c *^receptor variants function as XMV receptors for all 3 isolates.

AKR6 MLV shows typical xenotropic host range; it fails to infect mouse cells, but can infect cells of heterologous species [14]. When tested on mouse, rat, and mink cells, AKR6 showed the same general pattern of infectivity as the wild mouse CAST-X virus (Table 2) and NZB-IU-6 XMV (not shown). However, while other mouse XMVs showed low but reproducibly detectable infectivity in E36 Chinese hamster cells, AKR6 showed no such infectivity. Because infection of hamster cells with most gammaretroviruses is blocked by glycosylation [28], we examined virus infectivity in E36 cells treated with inhibitors of glycosylation (Table 3), as well as in Lec8 cells, a hamster glycosylation mutant that lacks GlcNAc-transferase I (Table 4). The reduction of glycosylation in hamster cells by mutation or by exposure to inhibitors results in increased susceptibility to ecotropic MLVs (not shown) and XMVs (Tables 3, 4), but did not relieve resistance to PMVs as observed previously [28], or to Cz524 or CasE#1. Unlike other viruses with XMV host range, however, AKR6 did not infect inhibitor-treated E36 cells or Lec8 cells. The human-derived XMV, XMRV, shows the PMV-like restriction of AKR6 in hamster cells; XMRV does not infect Lec8 cells or inhibitor-treated E36 cells (Tables 3, 4).

**Table 3:** LacZ pseudotype titers of X/PMV gammaretroviruses on E36 Chinese hamster cells treated with inhibitors of glycosylation.

	**Log_10 _LacZ Pseudotype Titer^a^**						
	
**Inhibitor**	**CAST-X**	**AKR6**	**XMRV**	**Cz524**	**CasE#1**	**FrMCF**	**HIX**
-	1.1+/-0.5	0	0	0	0	0	0
DMM	2.4+/-0.3	0	0	0	0	0	0
2DG	3.5+/-0.3	0	0	0	0	0	0
CST	2.5+/-0.4	0	0	ND	0	0	0

^a^Measured as the number of cells positive for β-galactosidase activity in 100 ul of virus.

0, no positive cells in cultures infected with 0.1 ml of undiluted pseudotype stock. ND, not done. Experiment was done four times. Glycosylation inhibitors were added the day before pseudotype infection.

**Table 4:** Infectivity of X/PMV LacZ pseudotypes on hamster and ferret cells.

		**Log_10 _LacZ Pseudotype Titer^a^**		
		
**Virus Type**	**Virus**	**Lec8**	**E36**	**Ferret**
XMV	CAST-X	3.3+/-0.8	1.1+/-0.5	5.6+/-0.3
	XMRV	0	0	3.9+/-0.01
	AKR6	0	0	5.3+/-0.4
	NFS-Th1	4.1+/-0.4	1.3+/-0.2	5.5+/-0.4
	NZB-IU-6	4.0	0.3+/-0.2	5.2+/-0.5
				
PMV	HIX	0	0	4.7+/-0.4
	FrMCF	0	0	5.4+/-0.4
				
X/PMV	CasE#1	0	0	5.1+/-0.3
				
X/PMV	Cz524	0	0	5.7+/-0.2
				
A-MLV	4070A	3.9+/-0.4	3.7	3.0+/-0.4^b^

^a^Measured as the number of cells positive for β-galactosidase activity in 100 ul of virus.

0, no positive cells in cultures infected with 0.1 ml of undiluted pseudotype stock. ND, not done. Experiment was done four times. Glycosylation inhibitors were added the day before pseudotype infection.

^b^Ferret cells show a 100-fold reduction in susceptibility to A-MLV compared to mink lung cells.

### CasE#1

CasE#1 efficiently infected *M. dunni *cells (*Xpr1*^*sxv*^) and *M. pahari *cells (*Xpr1*^*p*^) as well as rat and mink cells, but failed to infect hamster cells, NIH 3T3 (*Xpr1*^*n*^) and cells carrying *Xpr1*^*c *^(Table 2). Reduced infectivity of this virus in NXPR-S relative to *M. dunni *suggests it may be subject to *Fv1 *restriction. The overall pattern of CasE#1 infectivity is distinct from that of the XMVs, PMVs and Cz524.

### XPR1 determinants for X/PMVs

**Figure 3:**
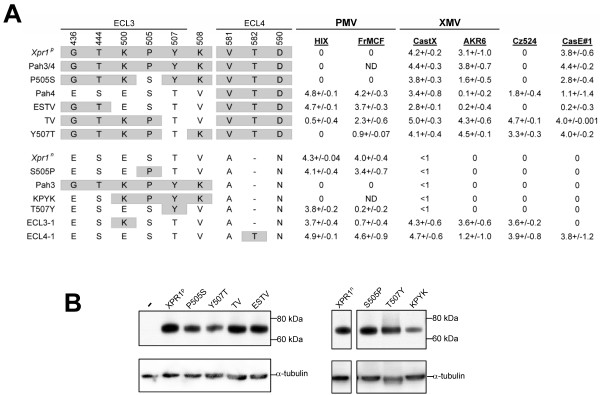
**Analyses of E36 cells**. Panel A. Susceptibility of E36 hamster cells expressing different *Xpr1 *receptors to LacZ pseudotypes of X/PMVs. Receptor genes cloned from NIH 3T3 cells (*Xpr1*^*n*^) and *M. pahari *cells (*Xpr1*^*p*^) were tested along with the indicated chimeras and mutants. Titers represent the averages of 3 or more experiments and are given as the number of LacZ positive cells/100 μl with SD. E36 cells show trace infectivity with CAST-X (<1). Panel B. Western blot analysis of the expression of E36 cells transfected with the indicated *Xpr1 *mutants. Expression was detected using an anti-V5 antibody (top). The lanes on the right were cut from the same photograph of a single Western blot.

To define receptor determinants for this panel of viruses, we tested 6 viruses for infectivity on E36 Chinese hamster cells expressing *Xpr1*^*n *^or *Xpr1*^*p *^as well as variants of the mouse XPR1 receptor (Fig. 3A). These transfectants included previously described chimeras between *Xpr1*^*p *^and *Xpr1*^*n *^and two *Xpr1*^*n *^mutations that independently introduce sensitivity to XMVs [6,7], namely E500K (mutant ECL3-1) and Δ582T (ECL4-1). We also generated a novel set of ECL3 substitutions made in *Xpr1*^*p *^or *Xpr1*^*n*^. Expression of the novel constructs in E36 cells was confirmed by western analysis (Fig. 3B).

Two *Xpr1 *variants reproduce the susceptibility pattern of *M. pahari*, that is, susceptibility to CasE#1 and all XMVs, but show resistance to PMVs and Cz524. Chimera Pah3/4 carries ECL3 and ECL4 of *Xpr1*^*p *^in an *Xpr1*^*n *^backbone demonstrating that the receptor determinants for XMVs and CasE#1 are in the ECL3 and ECL4 domains [7] (Fig. 3A). The same pattern of susceptibility is shown by the single ECL3 substitution P505S, although this change introduces an N-linked glycosylation site. The reciprocal change, S505P, made in *Xpr1*^*n*^, abolishes an N-linked glycosylation site, but does not alter the *Xpr1*^*n *^infectivity profile, that is, susceptibility to PMVs only. This suggests that residues at position 505 are not critical for PMV, XMV or CasE#1 entry. Western analysis shows that the P505S and S505P XPR1s show no obvious size differences suggesting that this glycosylation site is not utilized (Fig. 3B).

Reciprocal chimeras Pah4 and Pah3 contain, respectively, *Xpr1*^*p *^ECL3 (Pah3) or ECL4 (Pah4) in an *Xpr1*^*n *^backbone and are dramatically different receptors [7]. Pah3 is nonfunctional as a receptor for any of the tested viruses. Pah4 retains *Xpr1*^*n *^susceptibility to PMVs, but the combination of *Xpr1*^*n *^ECL3 and *Xpr1*^*p *^ECL4 introduces susceptibility to Cz524, CasE#1 and XMVs, although all inefficiently infect these cells except Cast-X.

The difference between the Pah3 and Pah4 chimeras suggests that the PMV receptor determinants are in ECL3; so we introduced substitutions at codon sites that distinquish ECL3 of *Xpr1*^*n *^and *Xpr1*^*p *^(Fig. 1). Mutant ESTV has substitutions in the 4 most C-terminal of these 6 sites in *Xpr1*^*p*^, and like Pah4, mediates susceptibility to PMVs. Making the reciprocal changes at these 4 sites in *Xpr1*^*n *^(mutant KPYK) results in loss of PMV susceptibility. Thus, some combination of residues at these 4 sites specifies the PMV receptor. Substitutions at positions 500, 507 and 508, all resulted in changes in the pattern of PMV susceptibility. Reciprocal substitutions were made at ECL3 position 507 in *Xpr1*^*p *^(Y507T) and *Xpr1*^*n *^(T507Y), and a double *Xpr1*^*p *^mutant carried K508V and Y507T. The two *Xpr1*^*p *^mutants acquired susceptibility to Cz524 and limited susceptibility to PMVs. T507Y retained susceptibility to HIX although infectivity with FrMCF was barely detectable. Finally, *Xpr1*^*n *^with E500K (mutant ECL3-1) is an efficient receptor for HIX, but a poor receptor for FrMCF. These results indicate that PMV infectivity is influenced by residues at the C-terminal end of ECL3, but that different PMVs rely on different residue combinations.

As shown previously, mutations E500K and Δ582T independently convert *Xpr1*^*n *^into a receptor for XMV [6]. Only one of these changes, Δ582T (mutant ECL4-1), generates a receptor for CasE#1 [7]. This mutation also produces a receptor for Cz524, results in reduced susceptibility to AKR6, but does not change susceptibility to PMV. In contrast, E500K (mutant ECL4-1) is efficiently infected by AKR6. Thus, K500 provides a more efficient receptor for AKR6 than does the T582 insertion.

None of the ECL3 mutations in *Xpr1*^*n *^introduces susceptibility to CasE#1, confirming that its primary receptor determinant is in ECL4, although as for AKR6, substitutions in ECL3 residues influence the efficiency of infection.

Susceptibility to Cz524 is introduced into *Xpr1*^*n *^by either of the XMV determinants, Δ582T or E500K, or by the Y507T and K508V substitutions in *Xpr1*^*p *^that also introduce some susceptibility to PMVs. However, other mutant receptors carrying these residues are not susceptible to Cz524, suggesting that Cz524 has additional requirements for entry. The 4 most efficient Cz524 receptors are also efficient XMV receptors that also mediate PMV infection, suggesting that Cz524 virus utilizes receptor determinants required by both PMVs and XMVs.

## Discussion

We defined 6 variants of X/PMV gammaretroviruses with different species tropisms on rodent cells, and identified critical residues on the XPR1 receptor that mediate their entry. We identified two tropism variants among the PMVs, broad host range MLVs that can infect mouse cells as well as cells of many other species such as human and mink. FrMCF, unlike the other PMVs tested here, is very poorly infectious on rat cells. There are also 2 variants among the XMVs, viruses originally identified by their failure to infect cells of the laboratory mouse; AKR6 and the human derived XMRV, have XMV infectivity patterns on mouse cells, but resemble PMVs in their inability to infect hamster cells after the removal of the glycosylation block to gammaretrovirus infection. The fifth and sixth variants are represented by CasE#1 and Cz524, wild mouse isolates that differ from each other and from XMVs and PMVs in their pattern of infectivity on rodent cells.

Examination of the infectivity of these viruses on hamster cells expressing mutated XPR1 receptors establishes that different critical residues mediate entry of these viruses. As determined previously, K500 in ECL3 and T582 in ECL4 independently mediate entry of XMVs [6,7]. These determinants are not, however, functionally equivalent, as T582 but not K500 can function as a receptor for CasE#1, whereas K500 but not T582 provides an efficient receptor for AKR6.

Residues at the C-terminal end of ECL3 are critical for entry of PMVs. PMV receptor function is reciprocally altered in *Xpr1*^*p *^and *Xpr1*^*n *^by substitution of the 4 most C-terminal of the residues that distinguish these receptors. Mutations at one of these sites, position 505 in an apparently unused glycosylation site, do not alter PMV susceptibility. Mutations at the other 3 sites, positions 500 and 507 in ECL3, and position 508 at the boundary of the transmembrane domain, alter PMV infectivity, but substitutions at these sites do not produce equivalent receptors for HIX and FrMCF PMVs. These observations, together with the ability of all PMVs but FrMCF to infect rat cells suggest that different PMVs have different receptor requirements.

Mutations in the PMV critical sites in ECL3 also reduce infectivity by the AKR6 XMV. This, together with the PMV-like failure of this virus to infect deglycosylated hamster cells suggests that AKR6 relies on some critical sites that form the PMV receptor determinant.

Cz524 is a novel wild mouse isolate that is only able to efficiently infect mouse cells carrying one of the 4 *Xpr1 *receptors, *Xpr1*^*sxv*^. Cz524 resembles XMVs in its ability to infect *Xpr1*^*n *^modified by E500K or the insertion of T582, but examination of the larger set of mutants indicates that neither of these substitutions is sufficient to produce a Cz524 receptor. The fact that this virus infects cells susceptible to both PMVs and XMVs is not surprising as the Cz524 RBD sequence combines features of XMVs and PMVs. The overall sequence closely resembles that of XMVs, but its VRA shows a 3 amino acid deletion where PMVs have a 4 amino acid deletion. This suggests that this VRA indel is important for receptor interactions. The Cz524 sequence and its unusual tropism also suggest that several regions of the envelope may contact the receptor [18] and that the cell receptor interface is constructed from both ECLs.

### Receptor-mediated resistance and interspecies transmission

The characterization of entry-based virus resistance factors has obvious importance for a broader understanding of how viruses spread and adapt to new hosts, and how natural populations adapt to retrovirus infections. Infectious XMVs and endogenous X/PMVs have been identified in Eurasian mice, and these mice have evolved two protective mechanisms that restrict infection at the level of entry. Receptors can be blocked by Env glycoprotein produced by endogenous retroviruses (ERVs), and ERVs with intact *env *genes have been linked to the resistance genes *Fv4*, *Rmcf *and *Rmcf2 *[29-31]. More commonly, resistance to retrovirus entry is due to polymorphic mutations in the cell surface receptor. The present study indicates that the sequence variations that distinguish the rodent XPR1 receptors can result in subtle differences in the efficiency of virus infection or complete resistance to specific X/PMVs. Additional functional variants of XPR1 and determinants for X/PMV entry may be identified by expanding this analysis to non-rodent species exhibiting different virus susceptibility profiles [[14]; CAK, unpublished observations], as recently shown by a recent analysis of human/mouse XPR1 chimeras [15].

Receptor-mediated virus restriction can result in the outgrowth of virus variants able to circumvent such blocks by adapting to receptor variation, by using alternative receptors or, as in the case of XMVs, using alternative receptor determinants on the same protein. The panel of variant viruses used in the present study were all the products of such adaptations and included naturally occurring mouse-derived isolates, the human-adapted XMRV, and HIX and FrMCF, variants adapted to cultured cell lines or laboratory-bred animals. These viruses differ from one another at multiple sites within *env*. Mutagenesis studies focusing on these RBD differences and other *env *regions implicated in receptor binding and/or fusion should provide further information on the critical residues involved in entry and the factors that limit or extend receptor usage.

Defining genetic factors that underlie resistance to mouse gammaretroviruses is important because retroviruses are capable of trans-species transmission, and retroviruses that cluster with mouse gammaretroviruses are widespread among vertebrates. Martin and colleagues [32] found MLV-related ERVs in approximately one-fourth of the vertebrate taxa and identified recent zoonotic transmissions from mammals to birds and from eutherians to metatherians. Infectious viruses resulting from transspecies transmissions have been isolated from koalas and gibbon apes [33-35]. One of the viruses used in the present study, XMRV, is an infectious MLV-related virus from human prostate cancer patients [16,17], and it should be noted that similar viruses have also been reported in cell lines derived from other human tumors [36]. It would not be surprising to find more examples of interspecies transmissions involving MLVs, since mice have a worldwide geographic distribution and all mammalian species tested have functional XPR1 receptors [[14]; CAK, unpublished observation]. Thus, the examination of the co-evolution of the XPR1 receptor and the X/PMVs should contribute to an understanding of the natural history of infectious pathogenic gammaretroviruses in their murine hosts and provide a foundation for the study of epizoonotic infections.

## Conclusion

We used six natural variants of the rodent XPR1 receptor to define six distinct host range types among naturally occurring X/PMVs. The 6 host range types include a novel cytopathic virus of wild mouse origin, termed Cz524, with an unusual XMV-like *env *gene. Among the previously described X/PMVs used for this analysis, we identified two species tropisms among PMVs, described the unique host range of wild mouse isolate CasE#1, and showed that the mouse AKR6 XMV and the human-derived XMRV differ from other XMVs in their inability to infect hamster cells. We used mutant *Xpr1 *genes to demonstrate that these six host range types have overlapping entry requirements defined by 5 critical amino acids in two extracellular loops, K/E500, T507, V508, T582. This functional polymorphism of the rodent XPR1 receptor is a consequence of the antagonistic interactions between co-evolving host and virus genes that generate substantial variation at the interaction interface.

## Methods

### Viruses, cells, mice and virus assays

CAST-X is a xenotropic MLV isolated in our laboratory from the spleen of a CAST/EiJ mouse [7]. The human xenotropic-related virus, XMRV [16,17], was kindly provided by R. Silverman (Cleveland Clinic, Cleveland, OH). Cz524 is a novel MLV isolated from the spleen of a CZECHII/EiJ mouse 2 months after inoculation with MoMLV. Other viruses are listed in Table 1 and were originally obtained from Dr. J. Hartley (NIAID, Bethesda, MD) along with 3 additional Friend PMVs: Fr-MCF-1, FrMCF A1807 and MCF-Fr Nx.

Susceptibility to X/PMVs was tested in various cell lines including *M. dunni *[37], NIH 3T3, mink Mv-1-Lu (ATCC CCL64), Rat2 (CRL-1764), Chinese hamster cells E36 [38] and Lec8 (CRL-1737), a cell line from the Asian species *M. pahari *obtained from J. Rodgers (Baylor College of Medicine, Houston), rat XC cells (CCL-165), and E36 hamster cells transfected with *Xpr1 *variants. Embryo fibroblasts were prepared from the progeny of crosses between CAST/Rp and NFS/N mice that were homozygous for *Xpr1*^*c*^; these cells are termed NXPR-C. NXPR-S embryo fibroblast cells were prepared from NFS/N-*Xpr1*^*sxv *^congenic mice [39]. CAST/Rp mice were obtained from R. Elliott (Roswell Park Cancer Institute, Buffalo, NY). CZECHII/EiJ mice were obtained from The Jackson Laboratory (Bar Harbor, Maine). NFS/N and congenic mice were bred in our laboratory.

### Pseudotype assay

LacZ pseudotypes were generated for all viruses by infection of the packaging cell line GP2-293 (Clontech, Mountain View, CA) that had been transfected with pCL-MFG-LacZ (Imgenex, SanDiego, CA) along with pMSCVpuro (Clontech) by J. Silver (NIAID, Bethesda, MD). Filtered media from the virus infected cultures contained a mixture of infectious virus and LacZ pseudotypes. Cells were infected with appropriate dilutions of these pseudotype virus stocks in the presence of 4-8 μg/ml polybrene. One day after infection, cells were fixed with 0.4% glutaraldehyde and assayed for β-galactosidase activity using as substrate 5-bromo-4-chloro-3-indolyl-β-D-galactopyranoside (X-Gal, 2 mg/ml; ICN Biomedicals, Aurora, Ohio). Infectious titers were expressed as the number of blue cells per 100 microliters of virus supernatant.

### Inhibitors of N-linked glycosylation

Cells were treated by various inhibitors of N-linked glycosylation as follows: deoxymannojirimycin (DMM, 100 ug/ml); castanospermine (CST, 100 ug/ml), and 2-deoxy-D-glucose (2DG, 25 mM). All inhibitors were obtained from SIGMA (La Jolla, CA). Inhibitors were added to cultures that had been seeded the previous day and were not removed when pseudotype virus and polybrene were added 18-24 hours later.

### Generation of mutants and chimeras

Seven novel mutant variants of the *Xpr1 *gene were generated using previously described clones of *Xpr1*^*n *^and *Xpr1*^*p *^[7]. The mutants KPYK and ESTV were made by exchanging fragments of the 2 receptors using primers 1F, 1R, 2F, 2R (Table 5). All other mutants were generated by mutagenesis PCR using QuikChange II XL Site-Directed Mutagenesis Kit (Stratagene, La Jolla, CA). All mutants were confirmed by sequencing.

**Table 5:** Primers used to generate XPR1 mutants.

**Mutant**	**Primer sequence (5'→3')**	**GenBank number**
KPYK, ESTV	1F: AAATCCAGATTTTGGCTGCTCAA (1315-1337) 1R: GCAGGCACTGGATGAAGCGAA (1583-1603) 2F: TTCGCTTCATCCAGTGCCTGC (1583-1603) 2R: AAGAGACCCCAGTCCATCTTGA (1799-1820)	NM_011273

S505P	F: CACGAAGAACAAAATCACCCTGACACCGTGGTGTTCT (1705-1741) R: AGAACACCACGGTGTCAGGGTGATTTTGTTCTTCGTG (1705-1741)	NM_011273

T507Y	F: AGAACAAAATCACTCTGACTACGTGGTGTTCTTTTA CCTGTGG (1710-1752) R: CCACAGGTAAAAGAACACCACGTAGTCAGAGTGATT TTGTTCT (1710-1752)	NM_011273

P505S	F: CACAAAGAACAAAATCACTCTGACTACAAGGTGTTC (1495-1530) R: GAACACCTTGTAGTCAGAGTGATTTTGTTCTTTGTG (1495-1530)	EF606903

Y507T	F: AGAACAAAATCACCCTGACACCAAGGTGTTCTTTTA CCTGTGG (1500-1542) R: CCACAGGTAAAAGAACACCTTGGTGTCAGGGTGATT TTGTTCT (1500-1542)	EF606903

TV	F: AGAACAAAATCACCCTGACACCGTGGTGTTCTTTTAC CTGTGG (1500-1542) R: CCACAGGTAAAAGAACACCACGGTGTCAGGGTGATT TTGTTCT (1500-1542)	EF606903

Underlined letters represent introduced base substitutions. Numbers in parentheses indicate the position of each primer in the indicated GenBank sequence.

The recombinant plasmids were transfected into E36 Chinese hamster cells. Stable transfectants were selected with geneticin (830 μg/ml), and the expression of the *Xpr1 *variants was confirmed by western analysis. Proteins were extracted from transfected cells with M-PER Mammalian Protein Extraction Reagent (Pierce, Rockford, IL). The expression vector used for XPR1 inserts a V5 epitope at the C-terminus; XPR1 expression was detected in western blots using anti-V5 antibody (Invitrogen) followed by goat anti-mouse IgG conjugated with HRP (Invitrogen). The membrane was then stripped and incubated with mouse anti-α-tubulin (Sigma, St. Louis, Mo) and goat anti-mouse IgG conjugated with HRP (Invitrogen).

### Cloning and sequencing of env genes

RNA was extracted from Cz524, AKR6 and FrMCF virus infected mink cells. The full-length 2.1 kb *env *gene of Cz524 and AKR6 and the 0.9 kb segment of the 5' end of the FrMCF *env *were amplified by RT-PCR, cloned into pCR2.1-TOPO and sequenced. Primer sequences available on request. One substitution in the leader sequence, P4S, distinguishes our AKR6 from GenBank No. DQ199948. The sequences of the *env *genes of Cz524 and FrMCF were deposited [GenBank:GQ375545 and GenBank:GQ420673].

## Competing interests

The authors declare that they have no competing interests.

## Authors' contributions

YY produced and analyzed the *Xpr1 *mutants and cloned the *env *genes for sequencing. YY and QL carried out pseudotype infectivity assays. CK designed the study and wrote the manuscript. All authors read and approved the final manuscript.
